# Is there a benefit to adding dexamethasone to levobupivacaine in ultrasound-guided adductor canal block following anterior cruciate ligament reconstruction?

**DOI:** 10.3389/fmed.2025.1696393

**Published:** 2026-01-13

**Authors:** Svetlana Sreckovic, Petar Vukman, Radmila Klacar, Ana Odalovic, Vesna Jovanovic, Miljan Bilanovic, Darko Milovanovic

**Affiliations:** 1 Centre of Anesthesia and Resuscitation, University Clinical Center of Serbia, Belgrade, Serbia; 2 Clinic for Orthopedics Surgery and Traumatology, University Clinical Center of Serbia, Belgrade, Serbia; 3 Medical School, University of Belgrade, Belgrade, Serbia; 4 Department of Orthopedic Surgery, University Clinical Hospital Bezanijska Kosa, Belgrade, Serbia

**Keywords:** adductor block, anterior cruciate ligament reconstruction, dexamethasone, multimodal analgesia, postoperative pain

## Abstract

**Background:**

Reconstruction of the anterior cruciate ligament (ACL) often causes severe postoperative pain. This study aimed to assess how dexamethasone affects the analgesic effectiveness of the adductor canal block (ACB), opioid use, and rebound pain after ACL reconstruction with a bone-patellar tendon-bone (BPTB) graft.

**Methods:**

This non-randomized prospective study analyzed a total of 160 patients, who were divided into two groups: the Dexamethasone + ACB group and the ACB group.

**Results:**

Within the first 24 h after surgery, there was no difference between the groups in the percentage of patients experiencing pain (73.75% vs. 85%; *χ*^2^ = 2.4433; *p* = 0.118) or in pain severity during activity (1.74 ± 0.97 vs. 1.59 ± 0.65; *p* = 0.779). During the first 48 h after surgery, no significant difference was observed in opioid use between the groups. Three patients in the non-dexamethasone group reported rebound pain (*χ*^2^ = 0.2564; *p* = 0.61), while blood glucose levels were significantly higher in the dexamethasone group (*χ*^2^ = 4.329; *p* = 0.037).

**Conclusion:**

The addition of dexamethasone to the local anesthetic during ACB after ACL reconstruction is not supported due to the lack of benefits related to postoperative pain levels and the associated increase in glucose levels.

## 1 Introduction

The anterior cruciate ligament (ACL) injury is one of the most common ligament injuries in the human body, causing anteroposterior knee joint instability that significantly impacts sports activities and daily life for individuals (1, 2). An ACL injury often requires surgical repair with autografts, such as hamstring tendons, the middle third of the patellar ligament (BPTB), and the quadriceps tendon. However, graft harvesting can result in considerable postoperative pain at the donor site (1, 3, 4).

A multimodal analgesia regimen combines various pharmacological and non-pharmacological strategies to provide adequate pain relief and support early rehabilitation for patients (5–10). Peripheral nerve blocks are part of this approach, offering effective pain control without causing muscle weakness, and facilitating early recovery (5, 10). After ACL reconstruction, the adductor canal block (ACB) is presented as an alternative to the femoral nerve block (FNB) (11). However, the success of nerve blocks depends on the duration of local anesthetics and the use of adjuvant drugs to extend their effects (5). Additionally, the resolution of the nerve block, combined with a sudden increase in pain, can lead to *rebound pain,* which typically occurs within 24 h of the nerve block, and can hinder further rehabilitation (5, 12–14).

Dexamethasone has long been used as an adjuvant in various surgical procedures, although doses for perineural administration vary. 4 mg is the most common dose and is associated with prolonged effects of long-acting local anesthetics (15, 16). However, its use should be carefully considered, especially when other medications like nonsteroidal anti-inflammatory drugs (NSAIDs) and paracetamol are included in a multimodal plan after ACL reconstruction (15–17). The study aims to assess the impact of dexamethasone on the analgesic effectiveness of the adductor canal block, opioid consumption, and rebound pain after ACL reconstruction.

## 2 Materials and methods

### 2.1 Patients and study design

**Figure 1:**
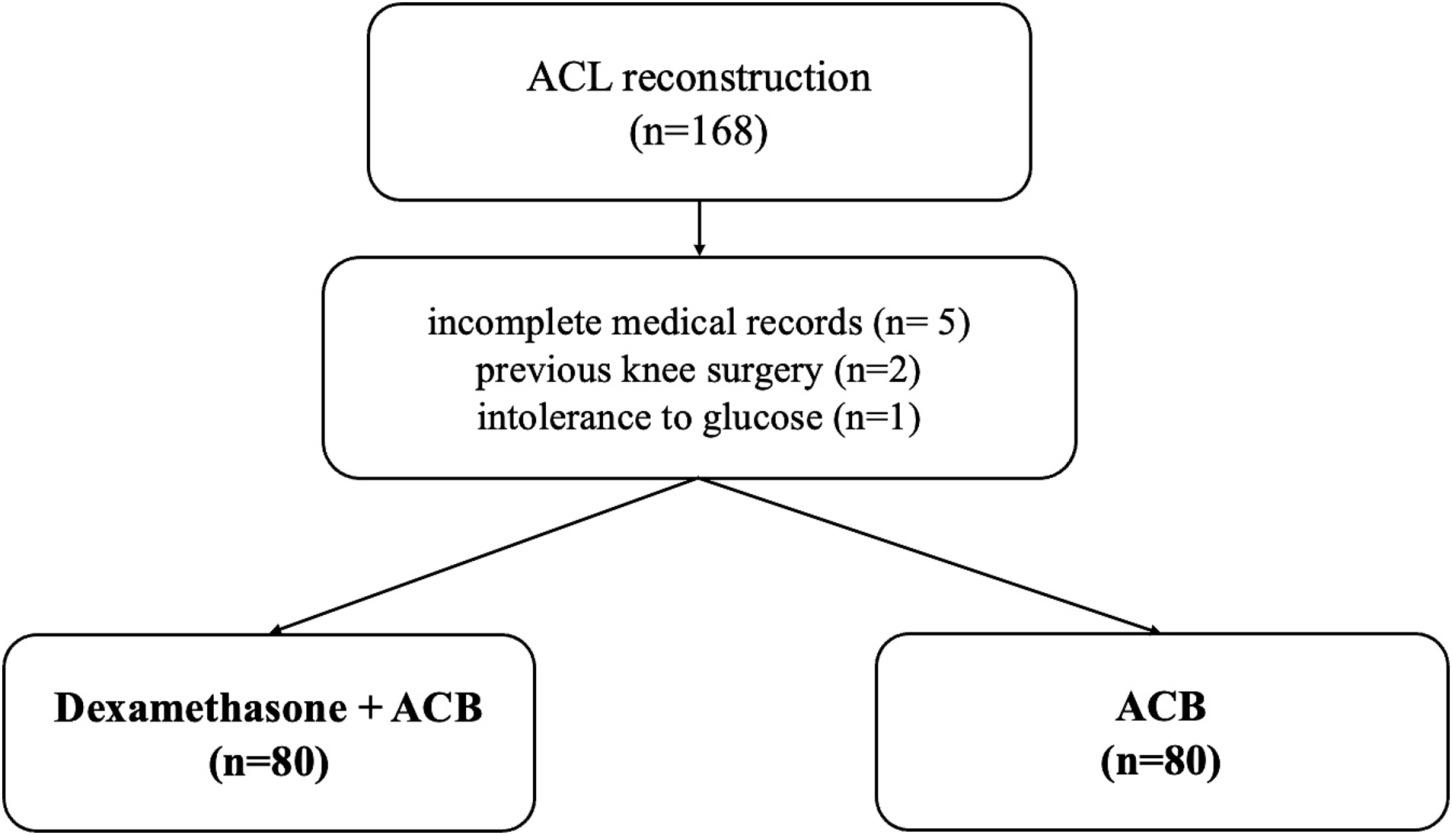
Patient selection.

This non-randomized prospective study involved 160 patients who underwent ACL reconstruction at a tertiary care hospital from February 2022 to February 2024. The study was conducted in accordance with the principles of the Declaration of Helsinki. Patients were included after receiving approval from the Ethics Committee of the University Clinical Centre of Serbia (No. 578/5 15.12.2021) and obtaining written informed consent (Figure 1).

The inclusion criteria were as follows: active athletes aged 15–45 with an isolated anterior cruciate ligament injury in the knee, who showed no clinical or radiographic signs of osteoarthritis, and underwent the same type of graft (BPTB graft). The exclusion criteria included incomplete medical records, previous surgery on either the injured or the opposite knee, intolerance to glucose or diabetes mellitus, opioid use within 30 days before surgery, and other conditions (such as substance abuse or mental illness) that could hinder recovery.

### 2.2 Intervention

ACL reconstruction was performed in a bloodless field using a tourniquet inflated to 300 mmHg under general anesthesia. Midazolam 0.05 mgkg^−1^, fentanyl 3 mcgkg^−1^, propofol 1.5–2.0 mgkg^−1^, and cisatracurium 0.2 mgkg^−1^ were used to induce general anesthesia. Anesthesia was maintained with the laryngeal mask and sevoflurane at a minimum alveolar concentration of 1. As a clinical standard, the postoperative analgesia regimen for all patients includes a combination of ACB and non-opioids.

Patients were divided into two groups based on whether they received dexamethasone with levobupivacaine for ACB: one group received 4 mg of dexamethasone, and the other did not. Patients were assigned to groups systematically (e.g., every other patient).

The ACB was performed with the patient lying down and the operated leg externally rotated. A linear ultrasound probe (15 MHz) was placed at the midpoint of the adductor canal to identify the sartorius muscle. A total of 15 mL of 0.33% levobupivacaine (± 4 mg of dexamethasone) was injected laterally to the femoral artery.

### 2.3 Postoperative outcomes

#### 2.3.1 Pain intensity

Pain intensity was measured at rest and during activity using the Numerical Rating Scale (NRS), which ranges from 0 (no pain) to 10 (worst pain imaginable), at various times during the first 24 h after surgery. Non-opioid medications, such as 1 g of intravenous paracetamol and 30 mg of intravenous ketorolac, were given alternately every 4 h as scheduled during the first 24 h post-surgery. If pain exceeded five, whether at rest or during movement, an opioid (morphine 1 mg IV) was administered until the pain subsided.

During the 24–48 h after surgery, patients received oral non-opioid medications (paracetamol, ketorolac) if their pain was up to five, or opioid medication (tapentadol 50 mg) if it was higher, whether at rest or during movement. Opioid use was expressed in morphine milligram equivalents (MME). Quadriceps strength was confirmed through manual muscle testing by the same surgeon before activity.

#### 2.3.2 Postoperative complications

The day after surgery, a fasting blood glucose level was measured. A value over 5.6 mmol/L was considered significant (18). Additionally, postoperative complications such as nausea, vomiting, itching, sleepiness, delayed wound healing, drainage, swelling, and rebound pain have also been reported. Rebound pain was defined as a pain intensity NRS score exceeding seven following the resolution of the nerve block (14).

### 2.4 Statistical analysis

The required number of subjects was estimated from preliminary experiments involving 20 patients, where the mean 24-h NRS scores for the two groups were 1.78 and 2.2, with standard deviations of 0.73 and 0.71, respectively. With *α* set at 0.05, a power of 90% (1−*β*), a two-tailed test, and an estimated dropout rate of 20.0%, the calculated sample size was 80 patients per group. As a result, this study enrolled 80 patients in each group.

The normal distribution was evaluated using the Normal Q–Q plot, histogram, and the Kolmogorov–Smirnov and Shapiro–Wilk tests. Data are shown as mean ± standard deviation (SD), median (minimum-maximum), and percentage (%) where applicable. All statistical tests were two-sided, with a *p*-value < 0.05 indicating significance. Differences between groups were assessed using the Pearson *χ*^2^ test, Fisher’s exact test, and independent two-sample *t*-test, depending on the parameters. For repeated measurements, the Bonferroni correction was applied. Data analysis was performed using the statistical software R (version 4.3.1 (2023-06-16 ucrt) - “Beagle Scouts”; Copyright © 2023 The R Foundation for Statistical Computing; Platform: x86_64-w64-mingw32/x64 [64-bit]) (available at: www.r-project.org; retrieved: 08/21/2023).

## 3 Results

There were no differences in patient characteristics—age, gender, BMI, or ASA status—between the two groups. The average age in both groups was 26 years. Nine females (11.25%) were in the Dexamethasone + ACB group, while seven females (8.75%) were in the control group (*χ*^2^ = 0.0694, *p* = 0.792). No significant difference in BMI was observed between the groups (*Z* = −0.2469, *p* = 0.803). All patients had ASA status I (Table 1).

**Table 1:** Patient characteristic.

Characteristics	Dexamethasone + ACB	ACB	*p*-value
Age (y)			
Mean (SD)	25.9 (6.37)	25.69 (4.86)	0.912
Median (range)	25 (15–44)	25 (17–40)	
Sex—*n* (%)			
Male	71 (88.75%)	73 (91.25%)	0.792
Female	9 (11.25%)	7 (8.75%)	
Weight (kg)			
Mean (SD)	79.97 (10.69)	77.7 (9.96)	0.658
Median (range)	80 (50–110)	76.5 (58–106)	
Height (m)			
Mean (SD)	1.79 (0.08)	1.78 (0.08)	0.653
Median (range)	1.78 (1.63–1.97)	1.795(1.61–1.98)	
BMI (kg/m^2^)			
Mean (SD)	24.85 (2.63)	24.29 (2.18)	0.803
Median (range)	24.58 (17.93–31.46)	24.28 (19.71–29.36)	
ASA physical status—*n* (%)			
ASA I	80 (100%)	80 (100%)	–
Total	80 (100%)	80 (100%)	–

BMI, body mass index; ASA, American Society of Anesthesiologists.

Two hours after surgery, a significantly higher percentage of patients in the ACB group reported pain (88.75% vs. 58.75%; *χ*^2^ = 17.078; *p* < 0.001), while there was no difference in pain intensity between the groups (Table 2). Additionally, 4 h post-surgery, patients reported mild pain (NRS < 3), with a notably higher percentage experiencing pain in the ACB group (88.75% vs. 75%; *χ*^2^ = 4.212; *p* = 0.04).

**Table 2:** Postoperative pain score.

Characteristics	In pain—*n* (%)			Pain (NRS)—mean (SD)		
	Dexamethasone + ACB	ACB	*p*-value	Dexamethasone + ACB	ACB	*p*-value
Pain after surgery, at rest						
2 h	47 (58.75)	71 (88.75)	**<0.001**	2.11 (1.24)	1.64 (0.75)	0.107
4 h	60 (75)	71 (88.75)	**0.040** ^*^	2.05 (1.47)	1.67 (0.47)	0.358
6 h	54 (67.5)	63 (78.75)	0.154	1.67 (0.89)	1.76 (0.89)	0.156
8 h	54 (67.5)	57 (71.25)	0.731	2.0 (1.34)	1.79 (0.41)	0.772
12 h	52 (65)	55 (68.75)	0.736	2.0 (1.49)	1.64 (0.49)	0.952
16 h	47 (58.75)	74 (92.5)	<0.001	1.65 (0.95)	1.47 (0.53)	**0.005** ^*^
20 h	51 (63.75)	59 (73.75)	0.232	1.65 (0.81)	1.58 (0.5)	0.234
24 h	48 (60)	45 (56.25)	0.748	1.68 (1.04)	1.6 (0.5)	0.741
Within 24 h	75 (93.75)	80 (100)	0.069	1.75 (0.74)	1.63 (0.26)	0.100
Pain during activity	59 (73.75)	68 (85)	0.118	1.74 (0.97)	1.59 (0.65)	0.779
Total	80 (100)	80 (100)	–	80 (100)	80 (100)	–

^*^p < 0.05.

Sixteen hours after surgery, 92.5% of patients in the ACB group reported significantly lower pain levels compared to those in the Dexamethasone + ACB group (Table 2).

During the activity, the percentage of patients experiencing pain (73.75% vs. 85%, *χ*^2^ = 2.4433; *p* = 0.118) and the pain intensity (1.74 ± 0.97 vs. 1.59 ± 0.65; *p* = 0.779) did not significantly differ between the groups (Table 2).

All patients received three intravenous doses of paracetamol and three doses of ketorolac within the first 24 h.

In the first 24 h after surgery, there were no differences between the groups regarding the number of patients who received opioids (*χ*^2^ = 1.558, *p* = 0.212) or the opioid dosage (*t* = 1.289, *p* = 0.199). One patient in each group, 24–48 h after surgery, required opioids at the same dose (Table 3).

**Table 3:** Postoperative opioid consumption.

Characteristics	Patients who needed opioids—*N* (%)			Dose of opioids (mg)—mean (SD)		
	Dexamethasone + ACB	ACB	*p*-value	Dexamethasone + ACB	ACB	*p*-value
Opioids consumption						
Within 24 h	1 (1.25%)	5 (6.25%)	0.212	5 (0)	6.0 (2.24)	0.199
24–48 h	1 (1.25%)	1 (1.25%)	0.477	5 (0)	5 (0)	–
Total	80 (100%)	80 (100%)	–	80 (100%)	80 (100%)	–

Within 24–48 h after surgery, the majority of patients in both groups received two doses of non-opioids (67.5% vs. 50%), with no difference in the number of doses between the groups (*χ*^2^ = 4.737, *p* = 0.192) (Table 4).

**Table 4:** Non-opioid use in the 24–48 h postoperatively.

Non-opioids	Dexamethasone + ACB	ACB
Number of doses, *n* (%)		
1	10 (12.5)	14 (17.5)
2	54 (67.5)	40 (50)
3	14 (17.5)	25 (31.25)
4	2 (2.5)	1 (1.25)
Total, *n* (%)	80 (100)	80 (100)

**Figure 2:**
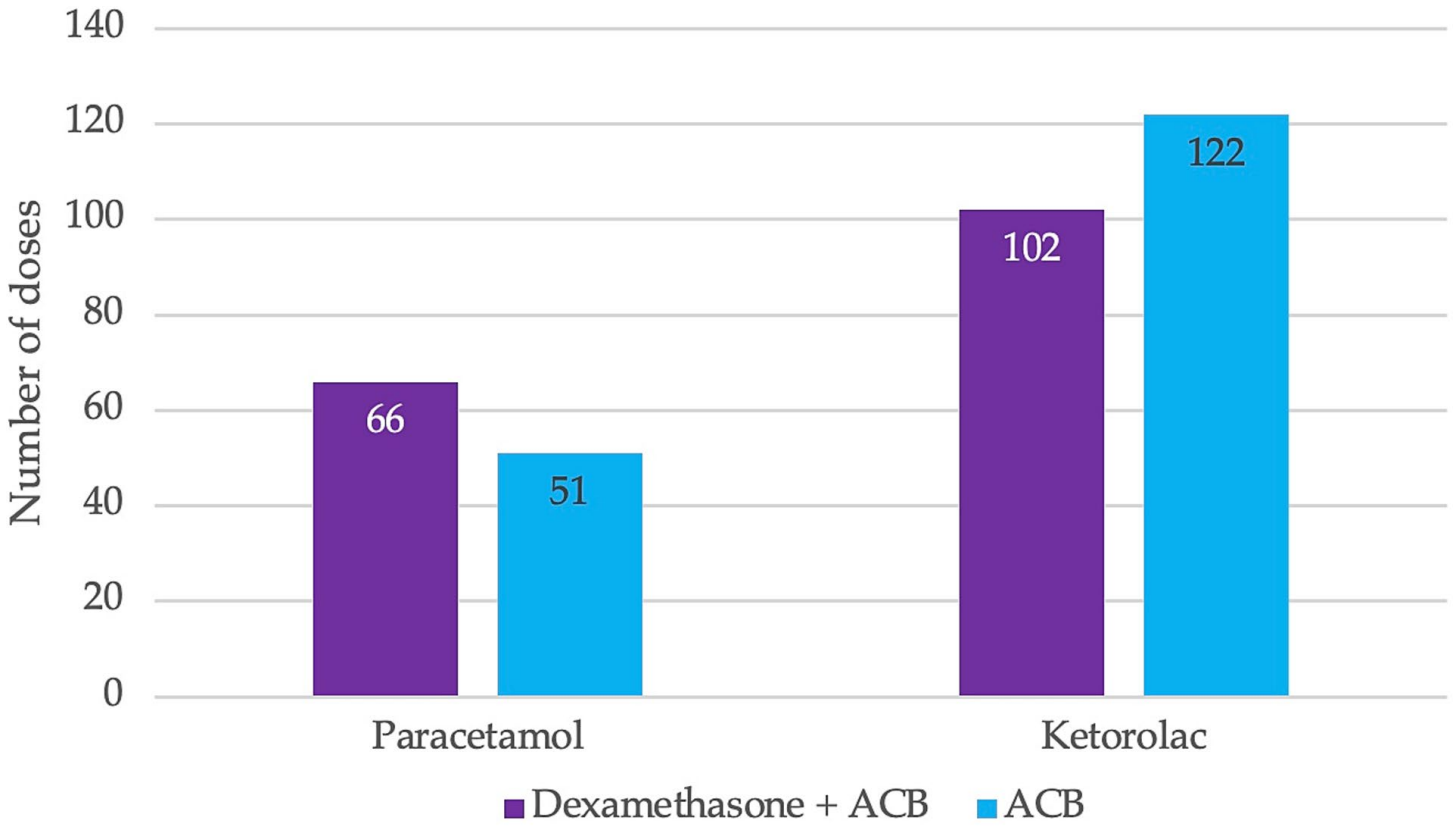
Total doses of non-opioids administered postoperatively between 24 and 48 h.

Paracetamol and ketoprofen were administered as pain relievers during the second 24 h after surgery, with no significant difference between groups (*χ*^2^ = 3.214, *p* = 0.073) (Figure 2).

Neither group experienced vomiting, urinary tract infections, delayed wound healing, drainage, or swelling. Three patients in the ACB group reported drowsiness (*χ*^2^ = 1.359, *p* = 0.244), while two experienced nausea (*χ*^2^ = 0.506, *p* = 0.477), and three reported rebound pain (*χ*^2^ = 1.359, *p* = 0.244), showing no significant difference compared to the second group. Elevated blood glucose levels were significantly higher in the group receiving dexamethasone (*χ*^2^ = 4.329, *p* = 0.037) (Table 5).

**Table 5:** Postoperative complications.

Postoperative complications	Dexamethasone + ACB (*n* = 80)	ACB (*n* = 80)	*p*-value
Rebound pain, *n* (%)	0 (0%)	3 (3.75%)	0.244
Nausea, *n* (%)	0 (0%)	2 (2.5%)	0.477
Drowsiness, *n* (%)	0 (0%)	3 (3.75%)	0.244
Elevated blood glucose levels, *n* (%)	6 (7.5%)	0 (0%)	0.037^*^

^*^p < 0.05.

## 4 Discussion

Our study’s findings showed that adding dexamethasone to the local anesthetic for ACB after ACL reconstruction, along with non-opioid scheduling, did not reduce postoperative pain levels. Patients reported mild pain in both groups at various times during the first 24 h after surgery. At 2 and 16 h post-surgery, pain intensity was unexpectedly higher in the Dexamethasone + ACB group based on the distribution of pain scores across groups. However, it remained below three on the NRS, with no clinical significance. Additionally, there was no difference in pain during activities or in opioid use between the two groups. However, adding dexamethasone significantly increased blood glucose levels.

In a meta-analysis, Yin et al. demonstrated that both FNB and ACB effectively reduce pain and morphine consumption after ACL reconstruction. They also found that ACB results in better quadriceps strength and range of motion (14). Furthermore, Hoope et al. indicated that although FNB decreases opioid consumption immediately after surgery, it has a less favorable safety profile compared to ACB, leading to a higher incidence of motor blocks and near falls (19). Performing ACB is crucial to block the saphenous nerve and the nerve to the vastus medialis simultaneously while avoiding a motor block and accurately identifying the “ideal” position in the middle of the canal (11). Administering a large volume of local anesthetic in the proximal section of the adductor canal can cause undesirable muscle weakness due to the spread of the anesthetic to the femoral nerve. Meanwhile, any spread into the popliteal fossa can result in weakness in the foot and lower leg (11).

Various drugs are used as adjuvants to local anesthetics to enhance the quality and prolong the duration of peripheral nerve blocks (15). Dexamethasone can be administered either perineurally or intravenously (15, 19). Both approaches effectively extend the duration of sensory blocks, reduce postoperative pain, and decrease opioid consumption compared to a placebo (21). Using 1–4 mg of dexamethasone, the duration of analgesia for long-acting local anesthetics was 505 (342–669) min, while with 5–10 mg, the average duration was 509 (443–575) min (15). Additionally, these findings were confirmed in a recent meta-analysis, which showed that 4 mg of perineural dexamethasone increased the average duration of analgesia for long-acting local anesthetics (16).

When comparing intravenous and perineural dexamethasone, perineural administration proved more effective. The sensory block lasted over 3 h, and postoperative pain was significantly lower in the perineural dexamethasone group (21). Ibrahim et al. (22) demonstrated that adding dexamethasone to bupivacaine in ACB significantly prolongs sensory block duration, the time until the first analgesic requirement, and patient satisfaction scores following ACL reconstruction Khatri et al. (23) administered dexamethasone in two intravenous doses of 10 mg, showing that pain after ACL reconstruction was significantly reduced both at rest and during walking. Consequently, the need for antiemetic and rescue analgesic medications decreased. The 6-month follow-up revealed no differences in range of motion, wound complication rates, or adverse side effects.

In our study group, neither group experienced vomiting, urinary tract infections, delayed wound healing, drainage, or swelling. However, three patients in the non-dexamethasone group reported feeling drowsy, two experienced nausea, and three reported rebound pain. In the dexamethasone group, blood glucose levels were significantly higher.

Katerenchuk et al. conducted a meta-analysis examining the effects of a single intraoperative dose of dexamethasone on blood glucose levels at various times during the first 24 h after surgery. The use of dexamethasone was associated with a significant increase in blood glucose levels, ranging from 0.37 to 1.63 mmol^−1^ (6.7–29.4 mgdL^−1^) at each time point. However, no differences were observed between subgroups based on diabetic status or dexamethasone dose, leading the authors to conclude that the observed increase was not clinically relevant (24). Furthermore, perineurally administered dexamethasone enters the systemic circulation and produces glucocorticoid effects (24, 25). Systemic absorption from local injections is sufficient to cause hyperglycemia and other steroid-related effects, with variability depending on tissue vascularity and steroid formulation (24, 25). Consistent with this, perioperative dexamethasone administration has been associated with detectable increases in blood glucose levels compared with control groups, even in patients without diabetes, suggesting an additive effect on stress-induced hyperglycemia (26).

In this study, patients receiving an adductor canal block with dexamethasone showed a statistically significant increase in blood glucose levels, whereas no such rise was observed in the control group without dexamethasone. Since both groups were managed under similar perioperative conditions and patients with glucose intolerance or diabetes mellitus were excluded, this difference indicates a connection between perineural dexamethasone and postoperative hyperglycemia. The findings emphasize that even perineural steroid administration can have systemic metabolic effects and should be closely monitored, especially in patients with glucose intolerance or diabetes mellitus. This warrants further investigation to confirm its potential clinical significance, despite its small magnitude and transient nature.

As part of the multimodal approach to pain management, combining peripheral nerve blocks with non-opioids may provide a promising strategy for reducing opioid use after surgery (5, 6). In our study, we used only paracetamol and ketorolac, and only 6.25% of patients required opioids at a 5 mg MME dose. Moutzouros et al. compare a multimodal non-opioid analgesic protocol (acetaminophen, ketorolac, diazepam, gabapentin, and meloxicam) to a standard opioid regimen (hydrocodone-acetaminophen) following ACL reconstruction. They demonstrated that a multimodal non-opioid pain protocol achieved effective pain control with minimal side effects and high patient satisfaction (27).

This study’s strength is in its precise examination of the effect of adding dexamethasone to the local anesthetic mixture, thereby enhancing the real-world relevance and clinical usefulness of its findings. It addresses a common but under-supported clinical practice. The assessment of glucose levels also offers an important safety perspective, helping to identify potential metabolic risks associated with dexamethasone use. Additionally, the study supports multimodal, non-opioid pain management, demonstrating that NSAIDs and paracetamol within an adductor canal block can reduce postoperative opioid requirements, underscoring its role in the ongoing effort to develop opioid-sparing protocols.

Our study’s limitations include its single-center design and potential selection bias. Moreover, several factors that can affect pain intensity, such as anticipated pain, fear of surgery, and anxiety, were not evaluated before surgery. Glucose levels were only recorded before and 24 h after surgery. Therefore, these findings need further validation through randomized controlled trials.

## 5 Conclusion

In conclusion, our study demonstrates that combining an adductor canal block with non-opioids effectively manages postoperative pain after ACL reconstruction with a BPTB graft. Adding dexamethasone to the local anesthetic is unsupported due to the lack of pain relief benefits and the increase in glucose levels, highlighting that even perineural steroid administration can have systemic metabolic effects and should be closely monitored, especially in patients with glucose intolerance or diabetes mellitus. Further research is needed to confirm its potential clinical significance, despite the small and temporary effects. Using various medications (NSAIDs and paracetamol) as part of a multimodal approach to the adductor canal block may effectively reduce opioid use following ACL reconstruction.

## Data Availability

The raw data supporting the conclusions of this article will be made available by the authors, without undue reservation.
